# Antimicrobial, antibiofilm, cytotoxicity, and substantivity of aged garlic extract against oral bacteria: an in-vitro study

**DOI:** 10.1186/s12906-025-05012-8

**Published:** 2025-07-16

**Authors:** Meghana Sunil, Bhaskar Kurangi, Suneel Dodamani, Marwa Khalil, Aditi Chopra

**Affiliations:** 1 Department of Periodontology, Manipal College of Dental Sciences, Manipal, Manipal Academy of Higher Education, Manipal, Karnataka, 576104 India; 2 KLE College of Pharmacy, KLE Academy of Higher Education and Research, Belagavi, 590010 India; 3 Dr. Prabhakar Kore Basic Science and Research Centre, KLE Academy of Higher Education and Research, Belagavi, JNMC Campus, Nehru Nagar, Belagavi, 590010 India; 4 Department of Oral Medicine, Periodontology, Oral Diagnosis and Oral Radiology, Faculty of Dentistry, Alexandria University, Alexandria, 21527 Egypt

**Keywords:** Periodontitis, Periodontal disease, Oral bacteria, Garlic, Herbal, Antimicrobial, Ayurveda

## Abstract

**Background:**

Garlic (*Allium sativum* L.*)* is a powerful antimicrobial, antioxidant, and anti-inflammatory agent. Aged garlic has more antioxidant and antimicrobial properties compared to fresh garlic. Garlic has been used for the treatment of many oral and periodontal diseases. However, the efficacy of aged garlic extract (AGE) against periodontal pathogens has never been explored. Hence, this in vitro study aims to assess the antimicrobial, antibiofilm, substantivity, and cytotoxic properties of AGE against key periodontal pathogens and oral tissues.

**Methods:**

The antimicrobial properties of the AGE were evaluated by assessing the minimal bactericidal concentration (MBC) and minimal inhibitory concentration (MIC) against *Actinomyces viscosus*,* Streptococcus salivarius*,* Fusobacterium nucleatum*,* Prevotella intermedia*,* Porphyromonas gingivalis*,* Aggregatibacter actinomycetemcomitans*,* and Tannerella forsythia* compared to doxycycline and chlorhexidine using the serial dilution method. The antibiofilm properties of AGE were checked for *A. actinomycetemcomitans*, and *F. nucleatum* was checked using the standard crystal violet staining assay. The cytocompatibility was checked against human-derived gingival and periodontal fibroblasts and modified oral keratinocytes using 3-4-5-dimethylthiazol-2-yl-2,5-diphenyltetrazolium bromide (MTT) assay. The substantivity of the extract was checked against chlorhexidine on the dentin surface from extracted tooth samples using an ultraviolet spectrophotometer.

**Results:**

The growth of *A. viscosus*,* F. nucleatum*, and *S. salivarius* was inhibited by AGE at 50 µg/ml. At 25 µg/ml, *P. gingivalis* and *A. actinomycetemcomitans* were inhibited. *P. intermedia* growth required a higher concentration of 100 µg/ml. At 25 µg/ml and 100 µg/ml, AGE showed bactericidal activity against *A. viscosus* and *P. intermedia*, respectively. The anti-biofilm assay showed that the percentage inhibition was 37.99% for *F. nucleatum* and 2.52% for *A. actinomycetemcomitans*. The cell viability of gingival fibroblasts (90%) and modified human keratinocytes (80%) was maintained by AGE at concentrations of 2.5 mg/ml and 5 mg/ml, respectively. The mean difference in substantivity for chlorhexidine and AGE at one minute was statistically significant (*p* = 0.0112).

**Conclusion:**

AGE was effective in inhibiting the growth of periodontal pathogens. However, its antimicrobial effects were not statistically significant when compared to doxycycline. AGE is biocompatible with gingival and periodontal ligament fibroblasts and has good substantivity to the dentin surface.

**Supplementary Information:**

The online version contains supplementary material available at 10.1186/s12906-025-05012-8.

## Introduction

Periodontitis is a chronic immune-inflammatory disease affecting soft tissues (gingival and periodontal tissues) triggered by the accumulation of microbial biofilm [1, 2]. The biofilm accumulating on the hard and soft tissues in the oral cavity is the prime etiologic factor for periodontal disease [3]. The most common bacteria in dental plaque associated with periodontal diseases include *Fusobacterium nucleatum*,* Tannerella forsythia*,* Aggregatibacter actinomycetemcomitans*,* Porphyromonas gingivalis*,* and Prevotella intermedia* [3–6]. Therefore, to manage periodontal disease, the first action required is the elimination of microbial plaque accumulation, which in turn regulates the immune-inflammatory response of the host [7].

The oral biofilm and calculus removal are primarily achieved by performing effective periodontal debridement (supragingival and subgingival scaling via hand or machine-driven instruments) followed by root surface decontamination and subgingival instrumentation [8]. However, studies have found that microorganisms can recolonize the teeth, gingiva, and periodontal pockets within 2–3 weeks following periodontal debridement [9–11]. Therefore, to effectively control the microbial load, many adjuncts to subgingival instrumentation, such as antimicrobials (antibiotics), anti-inflammatory agents, and antioxidants, are prescribed to achieve optimal oral hygiene [12, 13]. Among the antimicrobials, antibiotics are the most used adjuncts to scaling and root planing (SRP) to control the microbial load [14]. Antimicrobial agents like amoxicillin, metronidazole, ciprofloxacin, doxycycline, and chlorhexidine are some of the most common antimicrobial agents used for managing periodontal disease [15]. These antimicrobial agents are used either locally (in the form of gels, mouthwashes, pastes, patches, powders, and fibers) or systemically, as tablets, capsules, or powders [16]. However, with erratic and rapid usage of antibiotics, many of the microbes are no longer responding to these antimicrobials, including the gold standard chlorhexidine. Due to the development of resistance in many putative periodontal pathogens to these antimicrobials, research on many herbal or plant-based extracts or products to treat periodontitis is being done [16]. Numerous plant-based phytochemicals, including Tulsi, neem, green tea, curcumin, guava, goji berries, and pomegranates, are effective against periodontal pathogens [16–21]. Garlic and its derivatives have also been shown to have promising effects against periodontal pathogens.

Garlic (*Allium sativum* L.) is a highly promising anti-inflammatory and antimicrobial agent for oral and periodontal disease. Since ancient times, garlic has been used to treat many infectious diseases, including fever, cold, influenza, leprosy, typhoid, and botulism. This is linked to powerful anti-inflammatory and antimicrobial compounds present in garlic. Garlic contains many phytochemical compounds, mainly allicin, S-allyl cysteine, S − 1-propenyl cysteine, *S*-allylmercaptocysteine, phenols, and diallyl sulfide (DAS) have been proven to have potent antimicrobial and anti-inflammatory [23]. Garlic also contains ajoene, vinyldithiins, polyphenolic compounds, rutin, pyrogallol, quercetin, gallic acid, resorcinol, protocatechuic acid, and kaempferol, and enzymes (like allinase, peroxidase, and myrosinase) that can control microbial growth and have anti-inflammatory effects. Allicin, diallyl disulfide, and s-allyl cysteine are some of the potent compounds in garlic with broad-spectrum antibacterial, antifungal, and antiviral properties [21–24]. Aged garlic has been shown to have more antioxidant compounds like phenols compared to fresh garlic. Hence, it has stronger antimicrobial, anti-inflammatory, and antioxidant qualities compared to fresh garlic [23, 28]. The ageing process of garlic is done by naturally extracting the garlic extract and soaking it in a water/ethanol mixture for more than ten months at room temperature [25, 26]. The constituents and antioxidant qualities of the volatile compounds increase as garlic ages due to their reaction with hydrophilic compounds [26, 27]. The aging process is also known to enhance its antioxidant properties, increasing the diversity of garlic’s antioxidant compounds [25–30].

Garlic has shown excellent broad-spectrum antibiotic-like properties against many Gram-positive and negative bacteria such as *Staphylococcus aureus*,* Escherichia coli*,* Salmonella enteritidis*,* Listeria monocytogenes*,* and Bacillus cereus* [29–32]. Previous studies have reported mixed antimicrobial effects of garlic against a few oral bacteria [22, 32–43]. For example, Bakri et al. (2005) reported for the first time that garlic is effective against many oral bacteria, with minimum inhibitory concentrations (MIC) ranging from 17.8 to 1.1 mg/ml and minimum bactericidal concentrations (MBC) from 35.7 to 1.1 mg/ml. Garlic was able to inhibit the protease activity of *P. gingivalis* by 94.88%, thus demonstrating its efficacy in managing periodontitis [33]. Rao et al. (2014) compared the antibacterial effects of chlorhexidine and garlic against oral salivary microorganisms, discovering that the colonies of salivary microbes were significantly reduced in the 0.12% chlorhexidine group (positive control) (50 ± 4) compared to garlic extract (5%) (700 ± 200) [34]. Hard-neck garlic was also compared to soft-neck garlic against *Lactobacillus acidophilus* and *Streptococcus mutans*. The zone of inhibition against *S. mutans* and *L. acidophilus* was greater for hard-neck garlic (24 mm) compared to soft-neck garlic (18 mm) compared to chlorhexidine gluconate (17 mm) [35]. A study by Fani et al. (2007) also reported positive effects of garlic on *S. mutans* isolated from carious teeth, noting that out of 105 carious teeth examined, 28 (30.4%) were resistant to antibiotics. However, the antibiotic-resistant strains of *S. mutans* were sensitive to garlic extract, with a MIC value of 4 to 32 µg/ml [36]. Fresh garlic juice has also been reported to effectively inhibit oral anaerobic bacteria and restore the biological adaptation and attachment of fibroblasts, with an MIC for the anaerobic bacteria ranging from 1.64 to 1.51 µg/ml [37]. Borhan-Mojabi et al. (2012) [38], Velliyagounder et al. (2012) [39], Houshmand et al. (2013) [40], Hutomo et al. (2021) [41], and Bachrach et al. (2011) [42] tested the efficacy of garlic and its derivatives on the primary colonizers of oral biofilm (Lactobacillus, Streptococci species (*S. mutans*,* S. salivarius*,* S. sanguis*), *Actinomyces oris*, and *Prevotella. aeruginosa* species) and reported mixed results. Houshmand et al. found that garlic did not show any zone of inhibition against *S. mutans*,* S. sanguis*, and *S. salivarius*. However, at 5% concentration, a large zone of inhibition for *P. aeruginosa* and *Lactobacillu*s spp. was noted [40]. Bachrach et al. (2011) evaluated the antibacterial properties of allicin, a major antimicrobial compound in garlic, against oral bacteria associated with caries and periodontal disease and found that allicin at a concentration of 600 µg/mL was effective in inhibiting the growth of *Actinomyces oris*,* S. sobrinus*,* and S. mutans*. In the planktonic state, 300 µg/mL of allicin was effective in inhibiting the colonies of *F. nucleatum* and *A. actinomycetemcomitans*, and *P. gingivalis. P. gingivalis* showed the lowest sensitivity to allicin (2,400 µg/mL) and allicin could inhibit the proteases and gingipains enzymes of *P. gingivalis*. Allicin also demonstrated a good bactericidal effect on *S. mutans* colonies, although the effect decreased as the biofilm matured [42]. Aqueous and ethanolic garlic extracts have also beencompared against two key periodontal pathogens (*A. actinomycetemcomitans* and *P. gingivalis*), with aqueous garlic extract showing higher bacteriostatic activity against *P. gingivalis* than *A. actinomycetemcomitans* (MIC: 16.6 µL/mL). The anti-proteolytic activity against *P. gingivalis* was greater for the aqueous than the ethanolic extract [24]. Shetty et al. (2020) confirmed that when compared with guava extract, aqueous garlic extract exhibited a better zone of inhibition against *P. gingivalis* than ethanolic garlic extract and both aqueous and ethanolic guava extracts [43]. These in-vitro studies have tested and confirmed the antimicrobial properties of garlic against oral and periodontal pathogens [23].

However, to our knowledge, limited studies (one animal study in dogs by Takashashi et al. (2023) and one clinical study in humans by Zini et al. (2020)) have examined the effect of aged garlic for managing gingivitis and periodontitis [27, 44]. The clinical study by Zini et al. found that consuming 300 mg of aged garlic daily for 18 months significantly reduced the pocket depth, gingival inflammation, and levels of organosulfur compounds in the breath [27]. The animal study by Takashashi et al. also reported that feeding 18 mg/kg/day of aged garlic extract (AGE) for 8 weeks improved the gingival index, reduced the level of volatile sulfur compounds in exhaled air, and enhanced the enzyme activity of periodontal pathogens. Moreover, AGE increased the concentration of salivary cathelicidin, a key salivary peptide that aids in antimicrobial and oral innate immunity [44].

However, no study has yet assessed the antimicrobial and antibiofilm effects of AGE for oral bacteria. To our knowledge, this is the first study to investigate the effect of AGE on oral keratinocytes, gingival fibroblasts, and periodontal ligament fibroblasts. No study has yet explored the substantivity of AGE on tooth surfaces. Thus, addressing this research gap, our study aims to evaluate the antibiofilm, antimicrobial, cytotoxicity, and substantivity properties of AGE against periodontal pathogens along with its effect on gingival and periodontal tissues. This study will lay the foundation for future research where dentists, clinicians, and researchers can utilize aged garlic either locally or systemically to manage oral and periodontal diseases.

The objectives of the study were:

Primary objective

1. To assess and compare the minimum bactericidal concentration (MBC) and minimum inhibitory concentration (MIC) of the aged garlic, 0.2% chlorhexidine gluconate, and doxycycline and ciprofloxacin for the following oral bacteria: *S. salivarius*, *P. intermedia*,* A. viscous*,* P. gingivalis*,* A. actinomycetemcomitans*,* T. forsythia*,* and F. nucleatum.*

Secondary objectives

1. To determine the antibiofilm properties of the bridging species *F. nucleatum* and *A. actinomycetemcomitans* for AGE.
2. To evaluate the biocompatibility of AGE with gingival fibroblasts, periodontal ligament fibroblasts, and modified oral keratinocytes compared to chlorhexidine.
3. To evaluate the substantivity of AGE in relation to chlorhexidine on the dentin surface.

## Experimental methodology

The study was done at the Department of Periodontology, Manipal College of Dental Sciences, Manipal, in collaboration with the Department of Microbiology and Department of Pharmacy, KLE, Belgavi, Karnataka, India. The study was conducted from 2021 to 2022. The study was done after obtaining institutional ethical committee approval with IEC 74/2022. Written informed consent was taken from all participants who provided the premolar for the experimentation. The pure 100% AGE (Kyolic aged garlic extract) was obtained from ‘Wakunga Pharmaceutical Private Limited’, Japan. The details of the composition of the extract provided by the company are provided as supplementary file 1. The following experiments examined the antimicrobial properties, tissue compatibility, and substantivity of AGE.

### Minimal inhibitory concentration (MIC) and minimal bactericidal concentration (MBC)

The MIC and MBC for AGEs were determined for the following periodontal pathogens: *P. intermedia: ATCC 25,611; T. forsythia: ATCC 43,037; S. salivarius (Ss): ATCC 25,975; A. viscosus (Av): ATCC 15,987* (Himedia Private Limited, India). The serial dilution method was used for determining the MIC [24]. Brain Heart Infusion (BHI) broth (200 µl) was added to 10 microtiter tubes (2 ml each). 200 µL of the AGE was added to the first and second tubes. After that, it was progressively diluted from the second to the ninth tubes. The optical density value (OD value) of the bacterial culture with 0.5 McFarland standards was adjusted. 50 µl of the bacterial suspension was added to the 3rd to 8th test tube. The first and last tubes served as positive (Doxycycline) and negative controls. The MIC of the AGE that exhibited inhibition of bacterial growth was recorded [20]. The MBC was determined by plating the first five tubes at their MIC value and then incubating them for 24 h [28]. The MIC and MBC for two additional bacteria (*Fusobacterium* *nucleatum* and *Aggregatibacter actinomycetemcomitans*) were performed using the Resazurin method as described previously [29]. 96-well culture plates were and an equal quantity of microbiological media was prepared in specified wells, 100 µl was prepared. Further, AGE was added in 100 µl, after adding plant extract in the first well, serially dilute the extract to the requisite concentrations. Next, add 20 µl of inoculum of bacteria to each well, except the positive control. Add 20 µl resazurin solution to each well. Incubate 1 to 4 h at 37 °C. Record fluorescence using a 560 nm excitation / 590 nm emission filter set. The active metabolite reduces resazurin into resofurin, which is pink and fluorescent in colour. The concentration at which resofurin is just reduced to resofurin by colour change from blue to pink was taken as MIC.

### Antibiofilm assay

The antibiofilm assay was performed to assess the effect of aged garlic on preventing the formation of or disrupting the oral biofilms formed. Since microbes function differently compared to the single microbe colony, we tested the effect of aged garlic extract on the bridging species that help in primary and secondary colonizers in biofilm and key microbes for biofilm growth. Biofilm formation and inhibition were assessed using a standard crystal violet staining assay with modifications [29]. Oral bacterial isolates were first cultured in Luria-Bertani (LB) broth and incubated at 37 °C for 72 h to allow growth. The overnight culture was then sub-cultured for 12 h, followed by a 1:100 dilution in fresh LB medium to obtain the working bacterial suspension. A volume of 100 µL of the diluted suspension was dispensed into the wells of a sterile, flat-bottom 96-well polystyrene microtiter plate. The plates were incubated at 37 °C under static conditions for 24 to 72 h, depending on the strain, to permit biofilm formation. Post-incubation, the planktonic (non-adherent) cells were carefully removed by inverting the plate and aspirating the supernatant. Each well was then gently rinsed three times with sterile distilled water to eliminate residual non-adherent cells. Care was taken not to disturb the adherent biofilm layer. To stain the biofilms, 125 µL of 0.1% (w/v) crystal violet solution was added to each well, and the plate was incubated at room temperature for 15 min. Excess stain was then discarded, and the wells were rinsed three to four times with water until no free stain remained. The plate was air-dried for 12 h to fix the stained biofilms. Following drying, the bound crystal violet was solubilized by adding 125 µL of 30% (v/v) acetic acid to each well. After a 15-minute incubation, 125 µL of the solubilized dye from each well was transferred to a new microtiter plate, and the absorbance was measured at 570 nm using a microplate reader. The absorbance values correspond to the biofilm biomass. Each experiment was conducted in triplicate, and appropriate controls, including sterile medium blanks and untreated biofilm-positive controls, were included in all assays [30].

### Assessment of cytocompatibility via MTT assay

The cytotoxicity of AGE for gingival and periodontal ligament fibroblasts and modified human keratinocyte cells was assessed by performing the ‘3-4-5-dimethylthiazol-2-yl-2,5-diphenyltetrazolium bromide’ (MTT) assay. The cells were seeded in microtiter plates containing fetal bovine serum with the minimum essential medium (density = 1 × 10^5 cells/mL serum). The plate was incubated to allow for attachment for 12 h. The AGEs and control drugs (Chlorhexidine and mitomycin) at their MIC were added serially in three-fold dilutions and incubated in a 5% CO_2_ at 37 °C for 48 h. Subsequently, 5 mg/mL of MTT dye was added to each well plate and incubated for 4 h. The medium and MTT dye were then aspirated, and the crystals of formazan were dissolved with dimethyl sulfoxide. The degree of absorbance of the dye was determined at 570 nm. The cytotoxicity was calculated as follows: 1 - absorbance observed in the experimental well-plate / (absorbance observed in the control well-plate) × 100 [31].

### Assessment of substantivity

Human healthy premolars (*n* = 5 per group) extracted from individuals undergoing orthodontic treatment were collected after obtaining written informed consent. The crown was separated from the root in each tooth using a diamond bur and an airotor. The crown portion was sectioned into sizes of 2 × 2 mm each and was fixed with resin. The sections were submerged in either aged garlic gel or 0.2% chlorhexidine gluconate solution (Periogard-Colgate, India) for one minute. The sections were immersed in distilled water (1 mL), and substantivity was checked at 5 min, 30 min, and 360 min by obtaining an aliquot from the tube at each time interval. The samples were analyzed using an ultraviolet spectrophotometer [20]. The mean values of the MIC, MBC, and substantivity assay were calculated to obtain the average value for each group. The statistical analysis of the mean was performed using the one-way ANOVA test. Statistical significance was considered at *P* < 0.01.

## Results

### MIC and MBC

The results of the MIC showed that *S. salivarus* was sensitive at 50 µg/ml of the aged garlic extract (Table 1). *A. viscosus* and *F. nucleatum* were sensitive to the extract at a concentration of 12.5 µg/ml. *A. actinomycetemcomitans* required 25 µg/ml of aged garlic extract to inhibit its growth. It was also noted that *P. intermedia* showed minimum sensitivity to the extract and required 100 µg/ml. The MIC of *P. gingivalis* was 25 µg/ml. At 25 µg/ml, AGE was effective to kill the colonies of *A. viscosus* and *P. gingivalis.* At 100 µg/ml, bactericidal effect against *P. intermedia* was noted. The MIC and MBC of aged garlic were found to be comparable to chlorhexidine and higher than doxycycline against all the periodontal pathogens. The MIC value for the bridging species, *F. nucleatum*, and the virulent species, *A. actinomycetemcomitans*, was also checked against ciprofloxacin. The MIC for AGE was noted to be 12.5 and 25% concentrations against *F. nucleatum and A. actinomycetemcomitans*, respectively. Complete inhibition was noted with Ciprofloxacin (Supplementary file 2: Figures 1s and 2s, Supplementary Table 1s).

**Table 1:** Minimal inhibitory concentration of the aged Garlic extract compared to chlorhexidine and doxycycline- the results showed that all tested microorganisms were inhibited at 50 µg/ml, except *Prevotella intermedia*, which required a higher concentration (100 µg/ml). The minimum bactericidal concentration of the aged Garlic. The results showed that aged Garlic extract was bactericidal for *Actinomyces viscosus* and *Prevotella intermedia* at 25 µg/ml and 100 µg/ml, respectively, and bacteriostatic for *Fusobacterium nucleatum* and *Streptococcus salivarius*

Microorganism	Aged garlic extract		Chlorhexidine		Doxycycline	
	MIC (µg/mL)	MBC (µg/mL)	MIC (µg/mL)	MBC (µg/mL)	MIC (µg/mL)	MBC (µg/mL)
*Porphyromonas gingivalis*	25	25	8.5	16.6	0.2	0.2
*Aggregatibacter actinomycetemcomitans*	25	25	30.1	15.4	0.4	0.8
*Prevotella intermedia*	50	100	16	16	0.2	0.2
*Fusobacterium nucleatum*	12.5	100	15.6	20.8	0.2	0.2
*Actinomyces viscosus*	12.5	25	16	32	0.4	0.2
*Streptococcus salivarius*	50	100	16	16	0.4	0.4

**Fig. 1:**
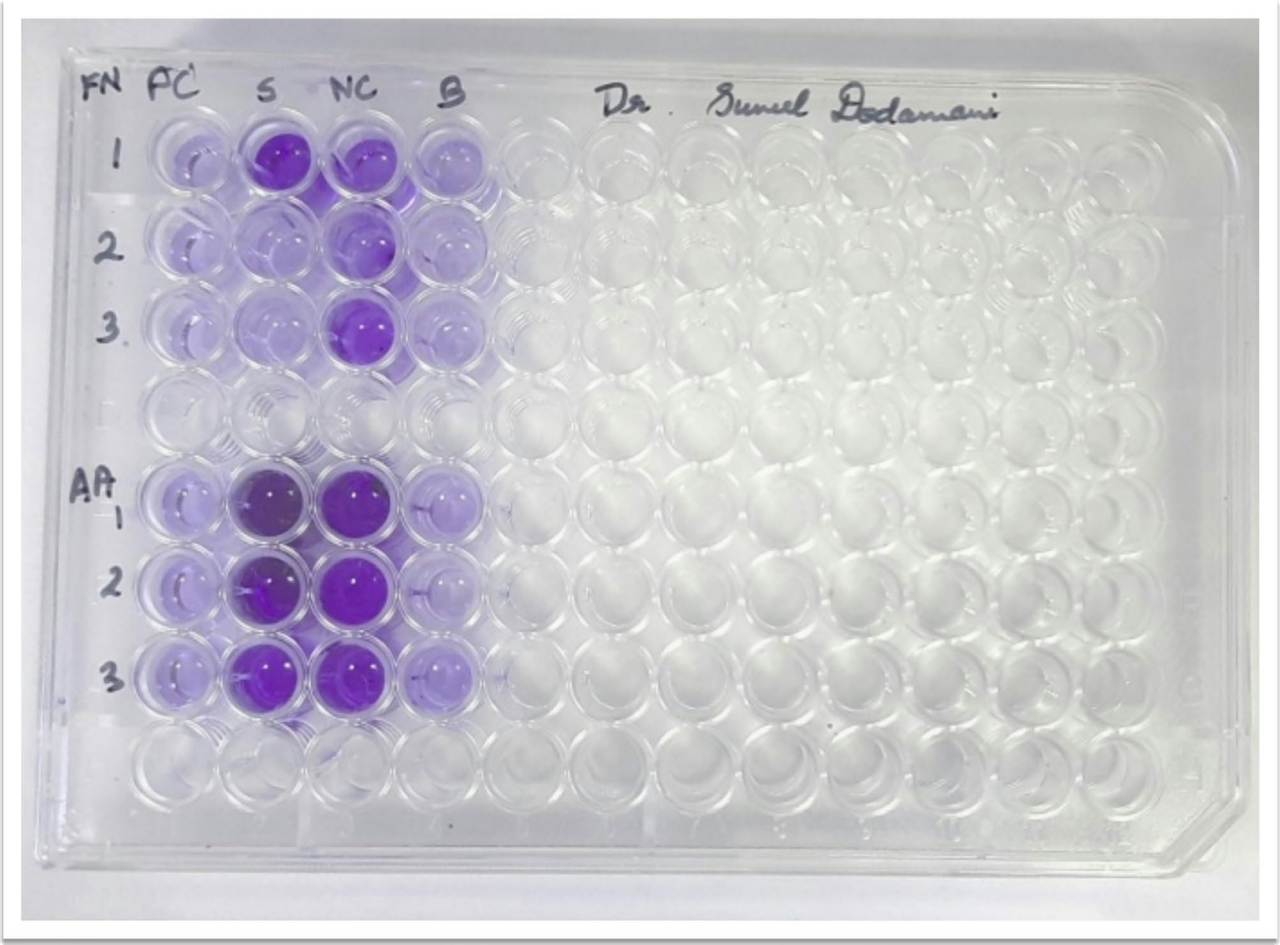
The anti-biofilm assay of aged garlic compared to ciprofloxacin for Fusobacterium nucleatum (FN) and Aggregatibacter actinomycetemcomitans (AA). Abbreviation: PC: Positive control (Ciprofloxacin); S: Standard (Aged garlic extract); NC: Negative control; B: Blank

**Fig. 2:**
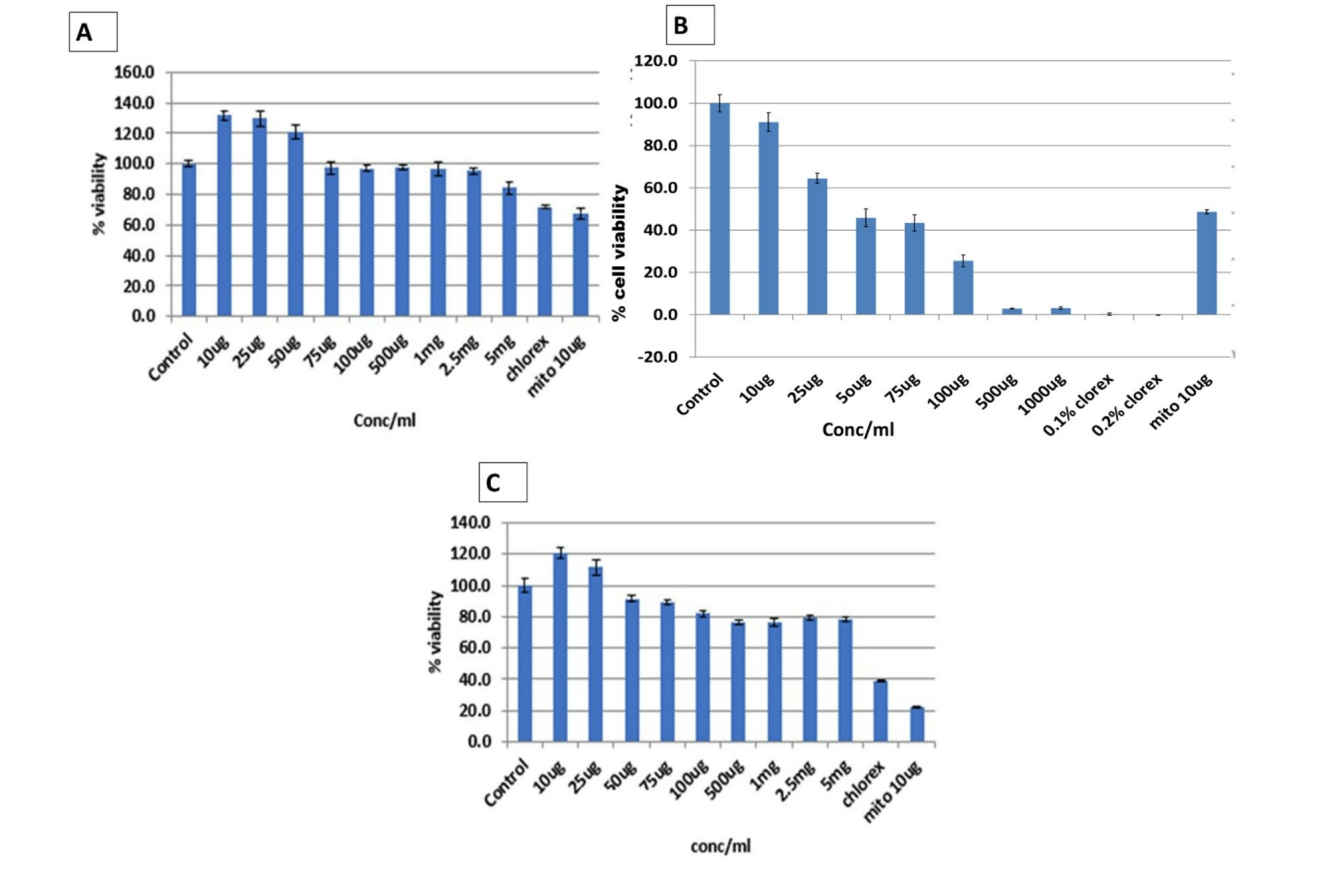
Graphical representations of cell viability for aged garlic extract compared to chlorhexidine and mitomycin: **A**) Almost 100% cell viability for the gingival fibroblast at 2.5 mg/ml compared to chlorhexidine, which shows around 70% cell viability. **B**): < 5% of cells were viable at 500 µg of AGE **B**) no periodontal ligament cell was found to be viable with 0.12% and 0.5 chlorhexidine gluconate; C: Around 80% modified culture human keratinocyte cell line (HaCaT) were viable at the concentration 5 mg/ml with AGE

### Anti-biofilm assay

The antibiofilm effect for the bridging species, *F. nucleatum*, was 37.99%. For *A. actinomycetemcomitans*, one of the most virulent bacteria associated with the aggressive nature of periodontal disease, the antibiofilm effect was 2.52%. Compared to the positive control (ciprofloxacin), the percentage of inhibition was found to be two times lower for *F. nucleatum* and 35.6 times lower for *A. actinomycetemcomitans* (Table 2; Fig. 1).

**Table 2:** The anti-biofilm assay of aged Garlic compared to Ciprofloxacin for Fusobacterium nucleatum and Aggregatibacter actinomycetemcomitans

Microorganism	Aged garlic extract	Ciprofloxacin	Only media
Fusobacterium nucleatum	0.896	0.288	1.445
Aggregatibacter Actinomycetemcomitans	3.363	0.349	3.45

### Cell proliferation assay

At a concentration of 2.5 mg/ml of AGE, approximately 90% of the gingival fibroblasts were viable. 80% of the keratinocytes remained viable at 5 mg/ml. Contrary to this, decreasing cell viability was noted for AGE in the periodontal ligament fibroblasts. Around 60% of cells were viable at a concentration of 25 µg, and < 5% of cells were viable at 500 µg and 1000 µg of aged garlic extract. It was also noted that almost no cells were viable at 0.12% and 0.2% concentrations of chlorhexidine (Fig. 2).

### Substantivity

The mean value for substantivity for chlorhexidine was higher than that of aged garlic extract. The mean absorbance for chlorhexidine at 1 min was 3.564 ± 0.113 (SEM = 0.050, *n* = 5). It reduced to 3.534 ± 0.027 (SEM = 0.012, *n* = 5) at 5 min but increased to 3.566 ± 0.053 (SEM = 0.23, *n* = 5) at 30 min and then reduced to 3.534 ± 0.027 (SEM = 0.012, *n* = 5) at 360 min. The mean absorbance for aged garlic gel increased from 3.084 ± 0.295 (SEM = 0.132, *n* = 5) at 1 min, 3.374 ± 0.194 (SEM = 0.087, *n* = 5) at 5 min, 3.616 ± 0.160 (SEM = 0.071, *n* = 5) at 30 min and 3.374 ± 0.194 (SEM = 0.087, *n* = 5) at 360 min. The mean difference in substantivity for chlorhexidine and aged garlic gel at 1 min (*p* = 0.0112) is statistically significant but not significant at 5 min (*p* = 0.159) and 30 min (*p* = 0.438). There is a statistically significant absorbance noted for aged garlic gel when compared to chlorhexidine at 360 min (*p* = 0.017) (Table 3).

**Table 3:** The mean absorbance of 0.2% chlorhexidine compared to aged Garlic gel is statistically significant at 1 min (0.011) and 360 min (*p* = 0.017) (*P* < 0.01 considered to significant)

Time Points	Aged Garlic gel	Chlorhexidine	*p*-value
	Mean ± SD		
1 min	3.084 ± 0.295	3.564 ± 0.11	0.011*
5 min	3.374 ± 0.194	3.534 ± 0.027	0.159
30 min	3.616 ± 0.160	3.566 ± 0.053	0.438
360 min	3.374 ± 0.194	3.534 ± 0.027	0.017*

## Discussion

Garlic and its derivatives have strong antimicrobial, anti-inflammatory, and antioxidant properties that are proven to be beneficial for managing periodontal disease [23, 44–49]. Previous studies have found garlic to be effective against many oral and periodontal pathogens [33–43]. However, the use of the aged form of garlic for periodontal disease has not been researched extensively. Since aged garlic has more antiglycation and antioxidant properties due to the presence of higher levels of phenols compared to fresh garlic, its use for managing periodontal disease is being advocated [49–51].

The present study is the first to assess the effect of AGE on both the key Gram-positive and Gram-negative oral bacteria. We found that AGE could inhibit the growth of *F. nucleatum and S. salivarius* at 50 µg/ml, with bactericidal effects against *A. viscosus and P. intermedia* at 25 µg/ml and 100 µg/ml, respectively. This indicates that AGE can prevent both early colonizers and bridging species in the oral biofilm. However, the antimicrobial properties were less than those of antibiotics (Doxycycline). These results are comparable to previous studies by Shetty et al. [24], Velliyagounder et al. [39], and Carrol et al. [52], where aged garlic was effective in reducing the growth and inhibiting the growth of *P. gingivalis* and *A. actinomycetemcomitans*. We also noted that AGE could inhibit both the early colonizers (*A. viscosus*, *S. salivarius*, and *P. intermedia*) and the bridging species (*F. nucleatum*). Due to its potential for causing severe infections, determining the MIC and MBC of different antibiotics against A. *actinomycetemcomitans* is critical for selecting appropriate treatment options. The antimicrobial effects on the *A. actinomycetemcomitans* are crucial for optimizing treatment strategies for the rapidly progressive nature of periodontal disease. The antibiofilm assay also confirms the ability of aged garlic to prevent the formation or disrupt the oral biofilms. Since microbes function differently compared to the single microbe colony, we tested the effect of aged garlic extract on the bridging species that help in primary and secondary colonizers in biofilm and key microbes for biofilm growth.

In addition to its strong antimicrobial properties, we also observed that AGE maintained the gingival and periodontal ligament fibroblast proliferation and vitality, unlike the gold standard, chlorhexidine. We also noted that AGE maintained over 90% viability of gingival fibroblasts and 80% of human keratinocytes at 2.5 mg/ml and 5 mg/ml concentrations. The percentage of cell viability for periodontal ligament fibroblasts was around 600% for 25 µg AGE compared to the percentage of cell viability with chlorhexidine, which was only 0.4 ± 0.25, and 0 ± 0.2% of the periodontal ligament cells were found to be viable with 0.12% and 0.2%. A similar study by Monika et al. (2023) compared the wound healing and anti-inflammatory properties of various herbal extracts (epicatechin, catechin, turmeric, garlic, neem, and pomegranate) by comparing their effects on the wound-derived fibroblasts. The authors found that at a concentration less than 100 µg/ml, all the herbs exhibited good cytocompatibility with the dermal fibroblasts. Garlic exhibited the highest cell viability among all the extracts [53]. Additionally, poor cell viability and cytotoxicity seen with chlorhexidine indicate its negative effects on gingival and periodontal ligament fibroblasts [51–55]. These findings are similar to the reports by Alleyn et al. [56] and Mariotti et al. [57], where chlorhexidine was found to inhibit the growth of the gingival fibroblasts at a concentration of 1300 µM (0.12%). Our study also noted comparable substantivity of AGE and chlorhexidine. The mean absorbance of chlorhexidine compared to aged garlic gel was statistically significant at 1 min (*p* = 0.011) and 360 min (*p* = 0.017), retained on the dentin surface. This demonstrates that when garlic extract is applied locally or sub-gingivally delivered, it will be retained on the surface. These properties have its potential to be used as a local drug delivery agent with sustained effect.

## Conclusion

Aged garlic effectively reduce and inhibits the growth of all periodontal bacteria tested along with demonstrating substantivity and cytocompatibility with keratinocytes, gingival fibroblasts, and periodontal ligament fibroblasts. These results prove that AGE possesses antibacterial properties against periodontal pathogens and holds potential for clinical applications in dentistry. AGE may serve as a local drug delivery method and be used as adjunct to periodontal therapy, particularly given the emergence of resistance among many oral bacteria to conventional antimicrobial agents [58]. However, further clinical studies are necessary to determine and confirm the potential of AGE as an adjunct to periodontal therapy for managing periodontal disease.

## Electronic supplementary material

Below is the link to the electronic supplementary material.

Supplementary Material 1

Supplementary Material 2

## Data Availability

The data can be made available upon reasonable request from the corresponding author.
