# Supplementary material for: Antimicrobial, antibiofilm, cytotoxicity, and substantivity of aged garlic extract against oral bacteria: an in-vitro study
Source: BMC Complement Med Ther. 2025 Jul 16;25:266. doi: 10.1186/s12906-025-05012-8 (PMC12265243; doi:10.1186/s12906-025-05012-8)
Supplement: Supplementary file 1 — Supplementary Material 1 [file 12906_2025_5012_MOESM1_ESM.doc]

Supplementary file 1

Aged garlic extract was characterized by post-column HPLC method using hexaiodoplatinate reagent which used for sulfur-specific detection as referenced previously by Matsutomo and Kodera , 2016 . According to analysis, 11 hydrophilic sulfur compounds were identified in aged garlic extract (Figure and table). Some of them identified were produced by aging process and these compounds, such as S-methylcysteine, S-allylcysteine, S-1-propenylcysteine and S-allylmercaptocysteine, are characteristic sulfur compounds in aged garlic extract (Ref 1 and other appropriate one).

Characterization of hydrophilic sulfur compound in aged garlic extract from post-column HPLC chromatogram is as follows:

| Rt | Mw | Area (%) | Compound |
| --- | --- | --- | --- |
| 4.12 | Not determined | 1.26 | Unknown |
| 8.61 | Not determined | 0.17 | Unknown |
| 10.60 | Not determined | 2.18 | Unknown |
| 13.44 | Not determined | 1.35 | Unknown |
| 24.16 | 149 | 4.11 | Methionine |
| 25.71 | Not determined | 0.49 | Unknown |
| 26.46 | 167 | 1.68 | *S*-Methylmercaptocysteine |
| 27.82 | Not determined | 0.58 | Unknown |
| 31.40 | 161 | 18.66 | *S*-Allylcysteine |
| 34.22 | 161 | 1.76 | *cis*-*S*-1-propenycysteine |
| 34.97 | 161 | 14.27 | *trans*-*S*-1-propenycysteine |
| 37.64 | 290 | 18.69 | γ-Glutamyl-*S*-allylcysteine |
| 40.96 | 175 | 20.97 | *S*-n-Butenylcysteine (Internal standard) |
| 41.99 | 193 | 6.84 | *S*-Allylmercaptocysteine |
| 49.25 | 322 | 6.99 | γ-Glutamyl-*S*-allylmercaptocysteine |

Chromatogram of hydrophilic sulfur compounds in aged garlic extract by post-column HPLC analysis. The chromatogram was monitored with 500nm absorbance.


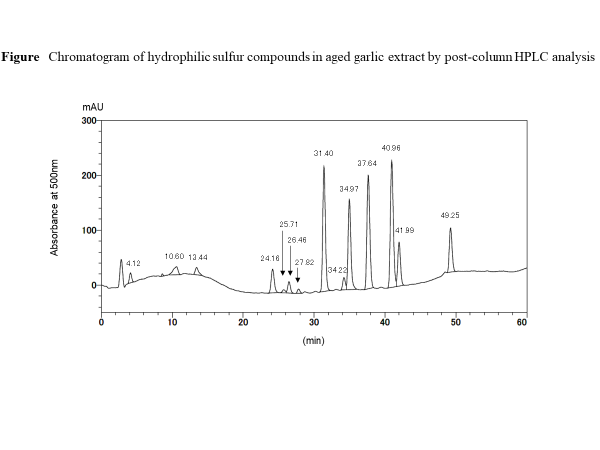


Reference: Matsutomo T, Kodera Y. Development of an analytical method for sulfur compounds in aged garlic extract using a post-column high performance liquid chromatography method with sulfur-specific detection. J Nutr 2016; 146(2):450S-455S.
