# Supplementary material for: Antimicrobial, antibiofilm, cytotoxicity, and substantivity of aged garlic extract against oral bacteria: an in-vitro study
Source: BMC Complement Med Ther. 2025 Jul 16;25:266. doi: 10.1186/s12906-025-05012-8 (PMC12265243; doi:10.1186/s12906-025-05012-8)
Supplement: Supplementary file 2 — Supplementary Material 2 [file 12906_2025_5012_MOESM2_ESM.docx]

**Dr. Prabhakar Kore Basic Science Research Centre, KLE Academy of Higher Education and Research**

**Report**

**Organism:** *Aggregatibacter actinomycetemcomitans* and *Fusobacterium nucleatum*

**Test performed:** Minimum inhibitory concentration and Minimum Bactericidal concentration

**
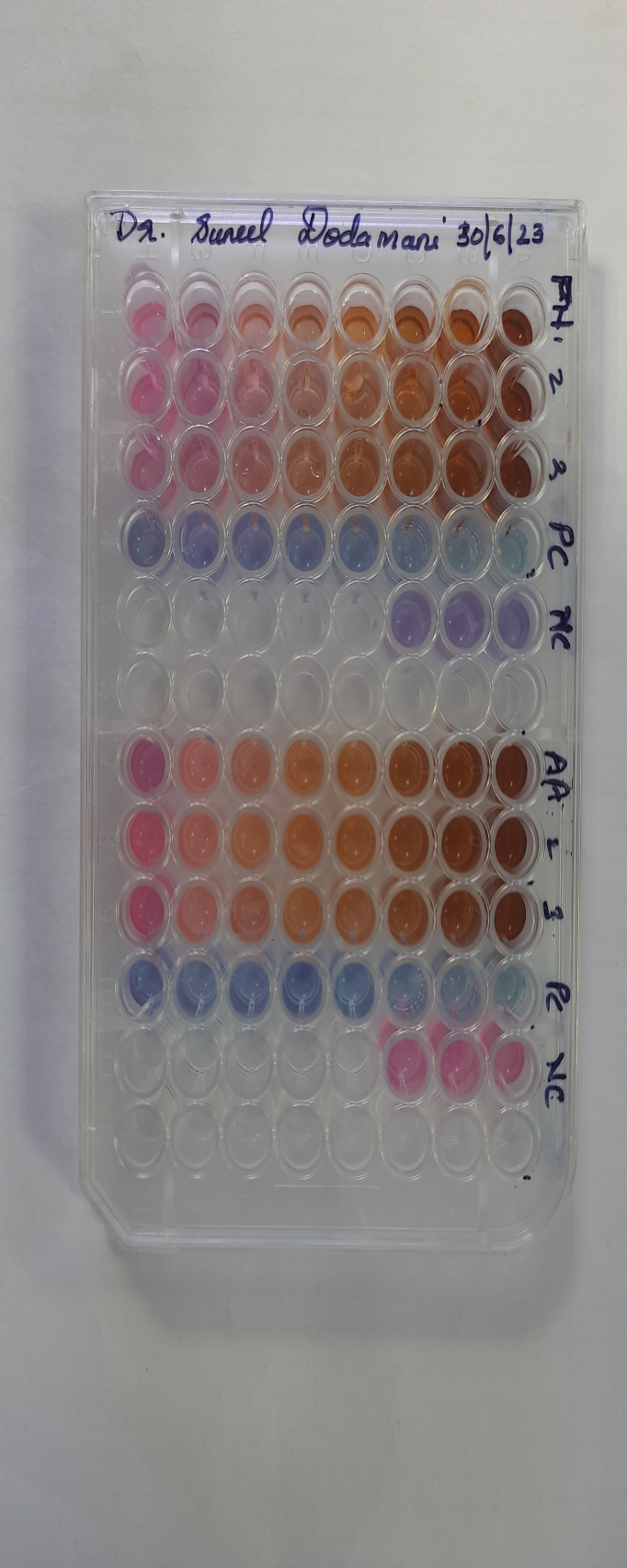
Media Used:** Brain Heart Infusion and Blood agar

**Supplementary Figure 1: MIC for *Fusobacterium nucleatum* and *Aggregatibacter actinomycetemcomitans* for aged garlic extract compared to Ciprofloxacin: A)**

**Supplementary Figure 2: MIC for Aggregatibacter actinomycetemcomitans (A) and Fusobacterium nucleatum (B) for aged garlic extract compared to Ciprofloxacin**

**
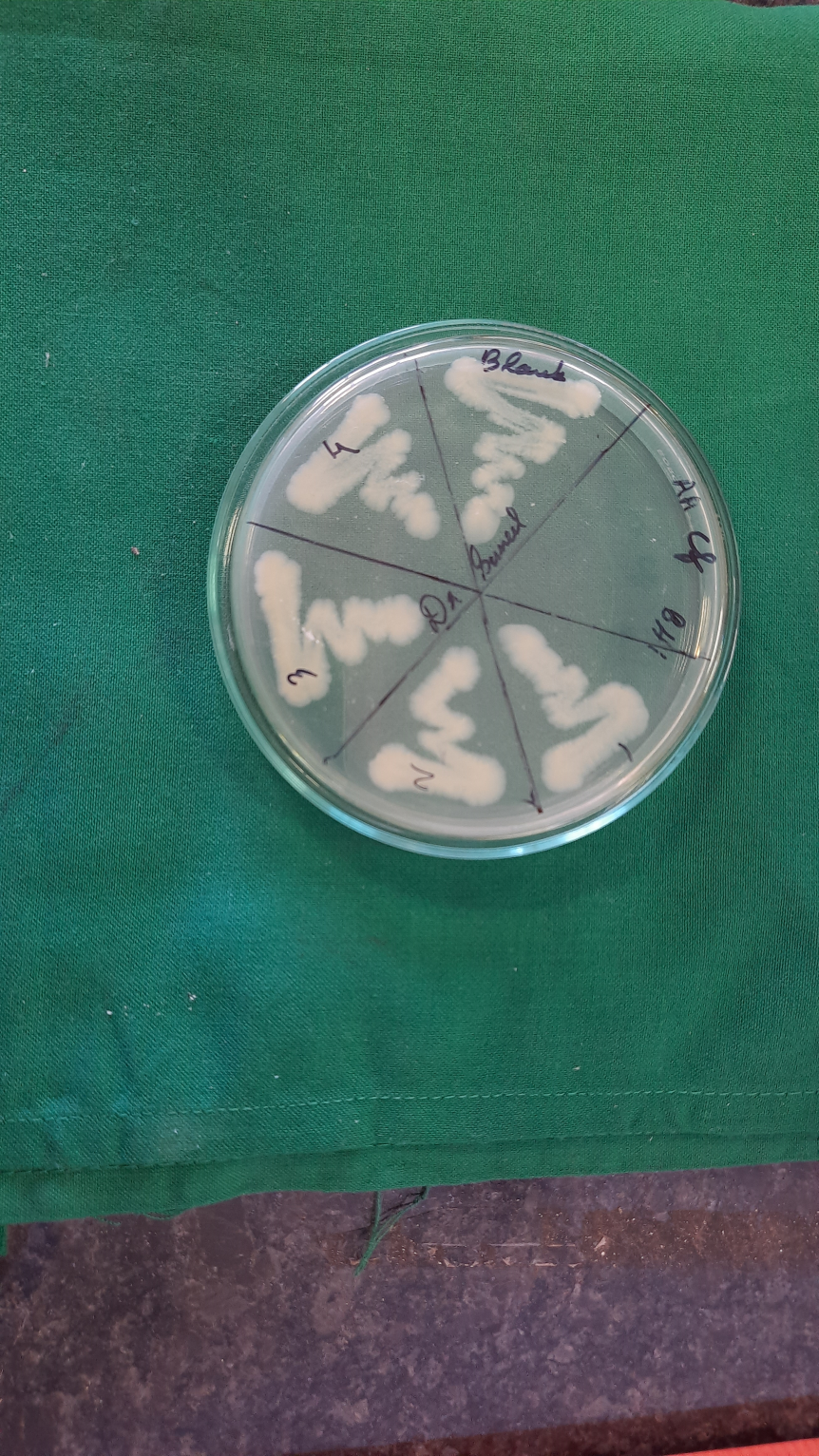

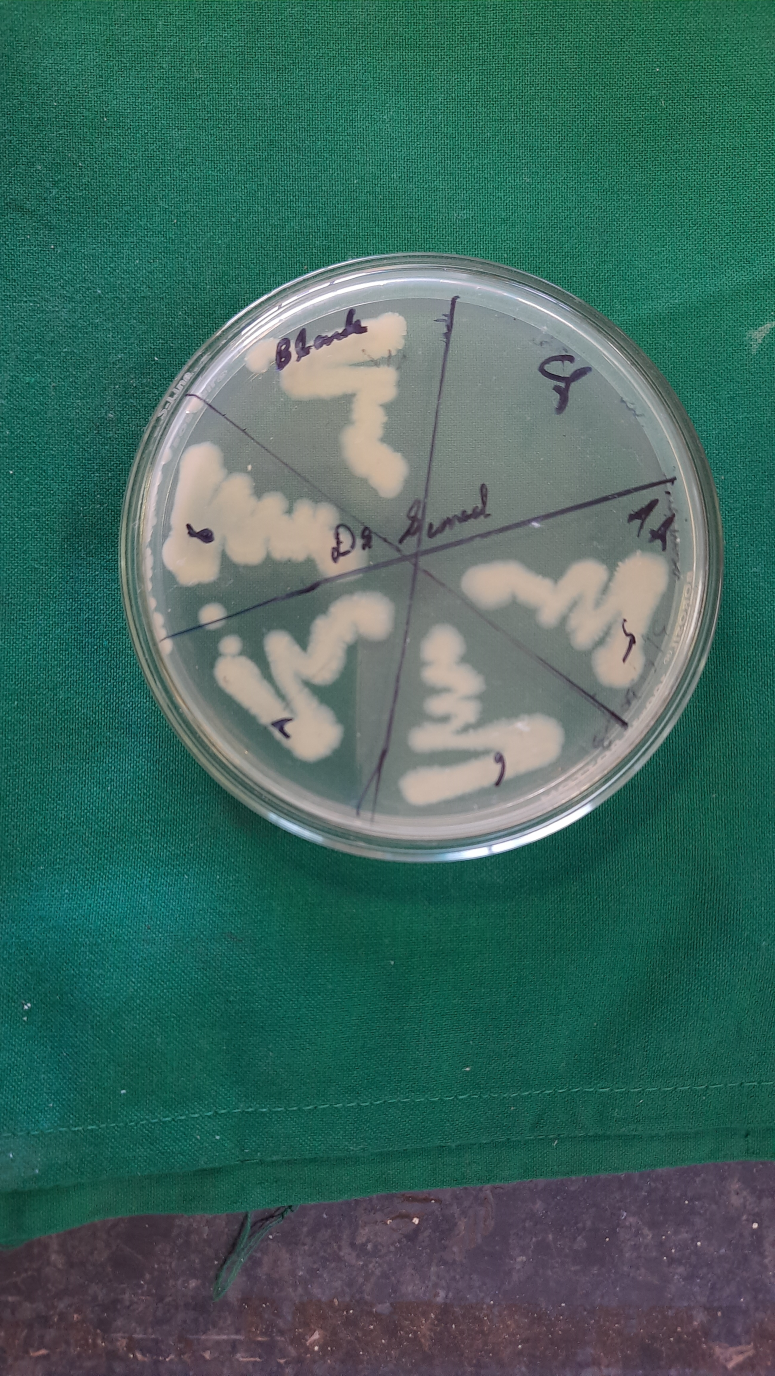
**

**A**

***Aggregatibacter actinomycetemcomitans***

**
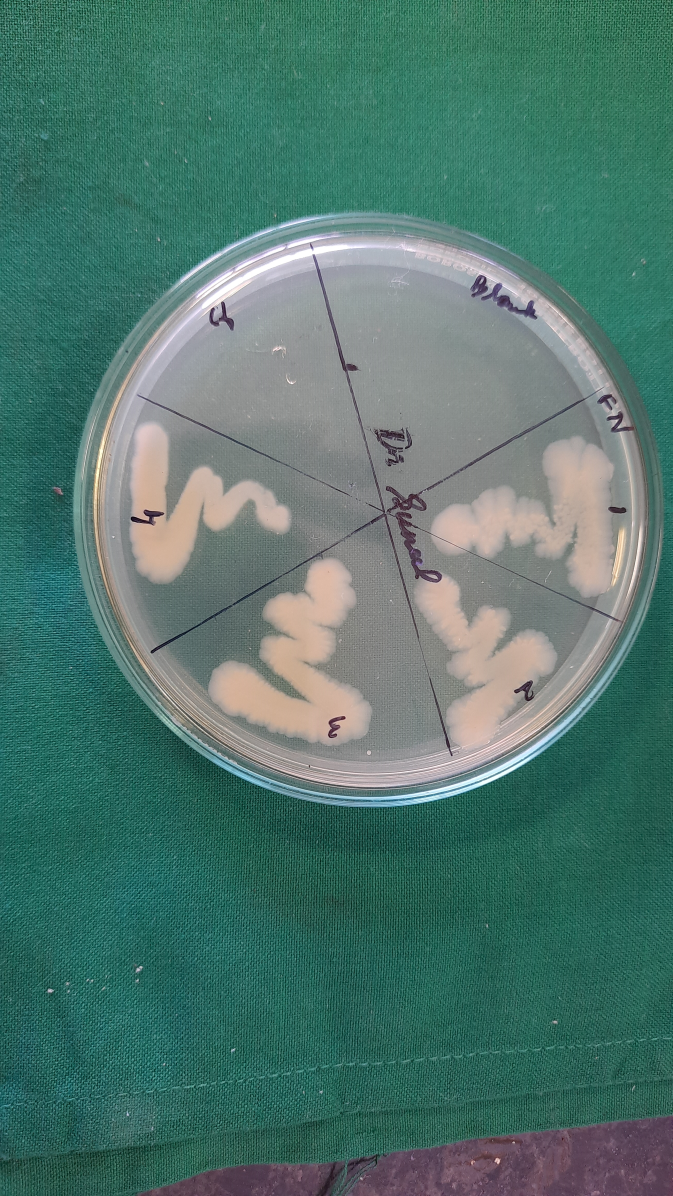

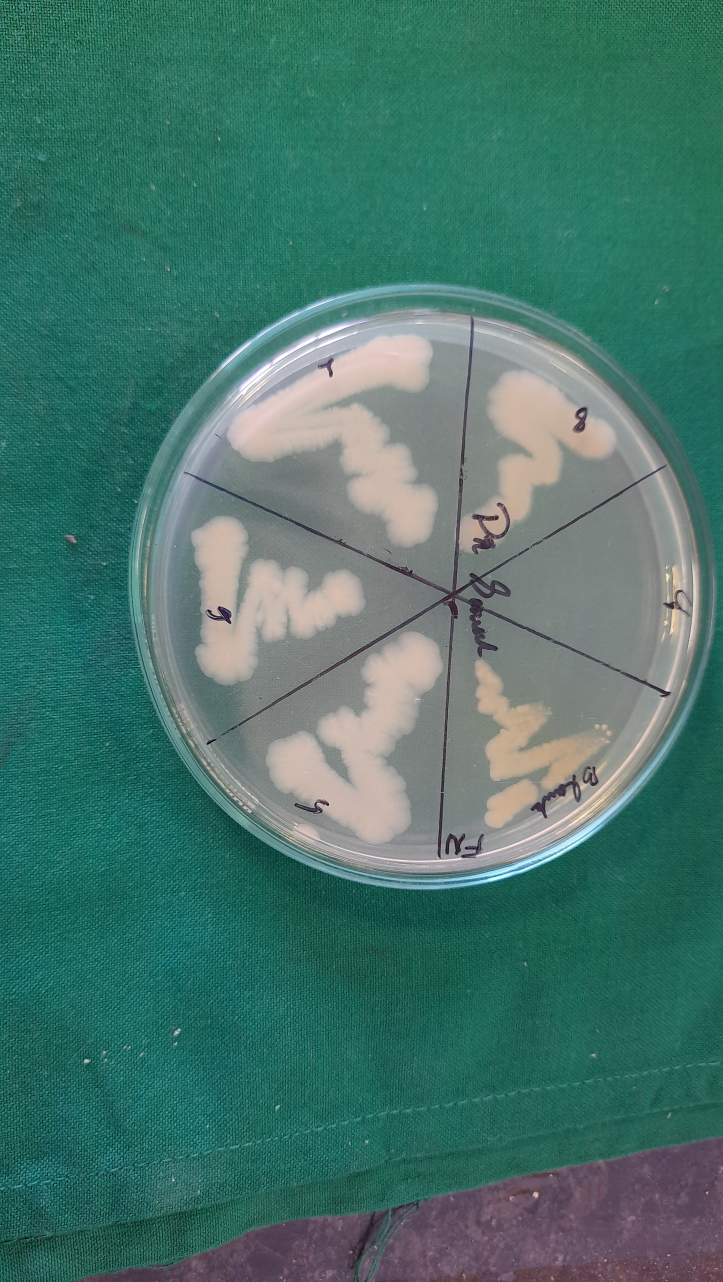
**

**B**

***Fusobacteriam nucleatum***

**Supplementary Table 1: MIC AND MBC for *Fusobacterium nucleatum* and *Aggregatibacter actinomycetemcomitans* for aged garlic extract compared to Ciprofloxacin**

| ***Fusobacteriam Nucleatum***  ***(Percent concentration)*** | | | **Ciprofloxacin** | ***Aggregatibacter actinomycetemcomitans***  ***(Percent concentration)*** | | |
| --- | --- | --- | --- | --- | --- | --- |
| **MIC** | **MIC**  **Average** | **MBC** | **MBC** | **MIC** | **MIC**  **Average** | **MBC** |
| 12.5 | 12.5 | G | NG | 25 | 25 | G |
| 12.5 |  | G | NG | 25 |  | G |
| 12.5 |  | G | NG | 25 |  | G |
